# Facial Expression Annotation and Analytics for Dysarthria Severity Classification

**DOI:** 10.3390/s26041239

**Published:** 2026-02-13

**Authors:** Shufei Duan, Yuxin Guo, Longhao Fu, Fujiang Li, Xinran Dong, Huizhi Liang, Wei Zhang

**Affiliations:** 1 College of Computer Science and Technology, Shanxi University of Electronic Science and Technology, Linfen 041000, China; 2 College of Electronic Information Engineering, Taiyuan University of Technology, Jinzhong 030600, China; 2024521515@link.tyut.edu.cn (Y.G.); flh087@163.com (L.F.); lifujiang10@126.com (F.L.); 2025520622@link.tyut.edu.cn (X.D.); 3 School of Computing, Newcastle University, Newcastle NE1 7RU, UK; huizhi.liang@newcastle.ac.uk; 4 Peking University First Hospital Taiyuan Hospital, Taiyuan 030032, China; zwwpa@126.com

**Keywords:** dysarthria, facial action units, keyframe labeling, Delaunay triangulation, graph convolutional networks, facial visual feature compensation

## Abstract

Dysarthria in patients post-stroke is often accompanied by central facial paralysis, which impairs facial motor control and emotional expression. Current assessments rely on acoustic modalities, overlooking facial pathological cues and their correlation with emotional expression, which hinders comprehensive disease assessment. To address this issue, we propose a multimodal severity classification framework that integrates facial and acoustic features. Firstly, a multi-level annotation algorithm based on a pre-trained model and motion amplitude was designed to overcome the problem of data scarcity. Secondly, facial topology was modeled using Delaunay triangulation, with spatial relationships captured via graph convolutional networks (GCNs), while abnormal muscle coordination is quantified using facial action units (AUs). Finally, we proposed a multimodal feature set fusion technology framework to achieve the compensation of facial visual features for acoustic modalities and the analysis of disease classification. Our experimental results using the THE-POSSD dataset demonstrate an accuracy of 92.0% and an F1 score of 91.6%, significantly outperforming single-modality baselines. This study reveals the changes in facial movements and sensitive areas of patients under different emotional states, verifies the compensatory ability of visual patterns for auditory patterns, and demonstrates the potential of this multimodal framework for objective assessment and future clinical applications in speech disorders.

## 1. Introduction

Dysarthria, a common sequela of stroke, results from central nervous system damage and is characterized by slurred speech and abnormal phonation [1,2]. Some patients also develop central facial paralysis, which manifests as asymmetry and reduced expressivity. Neuroimaging studies indicate that the internal capsule, corona radiata, and basal ganglia are involved in both articulation and facial motor control, with substantial anatomical overlap [3]. Nonverbal cues, including facial expressions and gestures, account for more than 65% of social communication [4]. Consequently, impaired speech and frequent facial paralysis in dysarthria significantly impact social functioning and mental health [5], with approximately half of all affected individuals experiencing depression or anxiety [6]. Additionally, restricted facial feedback further limits emotional expression. Despite these challenges, clinical assessments predominantly rely on subjective scales [7,8], often neglecting the influence of psychological issues and negative affect. Therefore, analyzing facial and acoustic differences during emotional tasks may provide a more objective evaluation of both disease severity and psychological state. Current research on dysarthria primarily focuses on acoustic indicators, since acoustic analysis is considered “a powerful complement to quantitative perceptual judgment” [9]. In recent years, acoustic features have generally been combined with deep learning techniques. For example, Joshy and Rajan [10] used acoustic features such as Mel-frequency Cepstral Coefficients (MFCCs) and identity vector (i-vector), along with models like deep neural networks (DNNs), Convolutional Neural Networks (CNNs), and Gated Recurrent Units (GRUs), to classify the severity of dysarthria in patients. Stumpf [11] employed a self-supervised Transformer network for multi-task modeling of speech signals to assess disease severity. However, for patients with moderate or severe dysarthria (especially those with aphasia), obtaining high-quality and semantically meaningful audio samples is almost impossible due to a significant decline in speech intelligibility [12,13]. As a result, it is difficult to fully capture the multidimensional nature of the pathology using acoustic features alone, which limits feature learning, model accuracy, and generalization.

It is worth noting that video modalities contain rich physiological information, such as mouth shape, lip movement, and facial muscle activity, which can effectively complement acoustic analysis in dysarthria assessment [14]. Motivated by this observation, recent studies have begun to explore multimodal approaches for dysarthria evaluation. For example, some works combine lip-related visual features with acoustic signals to improve recognition performance [15], while others analyze facial kinematics based on three-dimensional landmarks [16]. Despite these efforts, existing multimodal methods for dysarthria often focus on a limited set of specific articulatory organs (e.g., lips and jaw), while neglecting global compensatory facial movements and geometric structural changes that become increasingly prominent in moderate-to-severe cases. Beyond dysarthria-specific studies, the effectiveness of audiovisual fusion has also been demonstrated in related domains, such as monitoring neurodegenerative diseases and detecting coughs and sneezes associated with Coronavirus Disease 2019 (COVID-19) [17,18]. In the field of speech emotion recognition, various multimodal models have been proposed, including semantic-enhanced audiovisual networks, multimodal graph convolutional networks, and cross-modal alignment frameworks for speech reconstruction [19,20]. These advances collectively indicate that multimodal fusion is a powerful paradigm for modeling complex speech-related disorders. Therefore, for dysarthria, collaborative modeling of impaired speech and facial dynamics offers a promising direction to more comprehensively characterize pathological manifestations and improve the accuracy of automatic severity assessment.

Audiovisual modeling requires high-quality multimodal labels; however, existing dysarthria datasets are limited in both modalities and scale, particularly lacking data that combine audiovisual streams with emotion-elicitation tasks. The Whitaker database [21] contains isolated word pronunciations from six dysarthria patients, but the data are restricted to acoustic modalities, and the sample size is limited. The Torgo database [22] includes speech data and tongue articulation trajectory data from eight patients with cerebral palsy and seven healthy controls, but it does not incorporate video modalities. While the Dysarthric Expressed Emotional Database (DEED) database [23] provides audio–video bimodal information, it includes only four subjects, and the small sample size restricts the model’s generalizability; additionally, it does not address emotional expression tasks. Therefore, to investigate the relationship between emotional expression, facial movements, and the severity of post-stroke dysarthria, we constructed and annotated the THE-POSSD database [24], which includes data from 39 patients and 29 controls. This database integrates acoustic, glottal, and facial video data, along with emotional stimuli and psychological assessments. Using this resource, we annotate facial landmarks, action units, and key frames to facilitate subsequent dynamic expression modeling and feature extraction, to further evaluate the effectiveness of the video modality for disease classification.

Keypoint labeling is a crucial task in the field of computer vision, primarily used to identify key regions of interest in images or videos [25]. In facial analysis, keypoint labeling is a widely adopted standard that captures detailed information by locating the contour features of the chin, eyebrows, eyes, nose, and mouth. This process provides reliable data support for high-precision tasks, such as expression analysis and facial classification [26].

Facial action units (AUs) [27] are labeled based on a facial motion coding system that captures subtle changes in local muscles, such as those of the eyebrows, eyelids, corners of the mouth, and mandible, at the muscle level [28]. This system provides more accurate and physiologically interpretable quantitative indicators compared to overall expression features [29]. AUs are particularly sensitive to facial immobility in early Parkinson’s disease [30], and they are stable and accurate in emotion recognition and dynamic behavior modeling. Furthermore, some AUs correlate with valence and arousal, offering the potential to reflect subjective emotional states [28,31].

Keyframe-based annotation techniques capture facial dynamics, reduce redundant data, and improve analytical modeling efficiency. Common methods are based on image features [32], motion information [33], clustering [34], and deep learning [35]. However, automatic methods are highly dependent on labeled data, and existing dysarthria face samples are few and lack fine labels, which limits generalization and application; furthermore, it is difficult to acquire the comprehensive labeling of key points and AUs simultaneously. Thus, constructing a multi-level labeling system, which takes into account the multi-task requirements of facial key points, facial action units, and key frames, is the primary problem to be solved in this study.

At the level of facial feature extraction, facial key points exhibit non-Euclidean topology, which makes it challenging for conventional convolutional kernels to capture their relationships. Patients with stroke-related dysarthria often exhibit abnormal facial asymmetry and impaired motor sensitivity, highlighting the need for enhanced regional collaborative modeling [36,37]. Convolutional neural networks (CNNs) typically treat facial key points as independent pixels, which makes it difficult to effectively model the patient’s facial motion characteristics [36]. Studies have shown that Delaunay triangulation [38] can partition the face into multiple regions based on key points, thereby capturing spatial dependencies. In this case, an important challenge addressed in this study is how to effectively apply the Delaunay triangulation method to model the facial graph structure and extract the structural features of facial key points.

Finally, considering the input characteristics of audio–video dual-modal signals and the fusion of acoustic and visual multi-features, the third focus of this study is to verify the compensatory ability of the visual modality for insufficient acoustic expression, based on collaborative multimodal information modeling, and assess its effectiveness in the classification of dysarthria.

To sum up, this study proposes a three-level labeling method that leverages a pre-trained model and facial motion amplitude using the THE-POSSD dataset; constructs a multi-region facial mesh via Delaunay triangulation to enable the extraction and analysis of facial action units (AUs) and key points; and, finally, fuses multimodal information to enhance disease-classification performance and verify the visual modality’s compensation for acoustic deficits.

To present the research content clearly, this paper is organized as follows. Section 2 details the multimodal dysarthria dataset (THE-POSSD) constructed in this study, covering the data collection process and data composition. Section 3 elaborates on the multi-level annotation method based on pre-trained models and facial motion amplitude, aiming to alleviate the issues of missing annotations and data scarcity in existing datasets. Section 4 analyzes the statistical characteristics of facial action units (AUs) under different emotional states, constructs the facial action unit features (FAUF) and Delaunay-based Facial Landmark Graph Features (DFLGF), and validates their effectiveness through preliminary experiments. Section 5 proposes the Visual–Acoustic Compensatory Pathway Network (VACP-Net) and verifies the compensatory effect of the visual modality on acoustic features as well as the framework’s performance in dysarthria severity classification through comparative experiments. Finally, Section 6 and Section 7 provide an in-depth discussion of the experimental results and summarize the conclusions, limitations, and future research directions.

## 2. Dataset Introduction

The Taiyuan University of Technology-Taiyuan Central Hospital (TYUT-TCH) multimodal Emotional expression database of Post-Stroke Speech Dysfunction patients (THE-POSSD) is a Chinese multimodal emotional expression database of post-stroke motor dysarthria patients [24]. This database contains multimodal information such as acoustic, glottal, and video.

**Figure 1:**
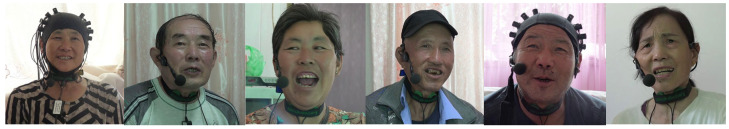
Patient facial data. All patients gave permission for the use of their images.

Video-modal data were used in this study. A total of 60 participants (35 with post-stroke motor speech disorder and 25 healthy controls) were enrolled. Given that patients are limited in emotional expression under different conditions, and surveys show that happiness, sadness, and anger are the most important communication emotions [25], four types of emotions were set for the dataset: happy, angry, sad, and neutral. Some patient video screenshots are shown in Figure 1.

The original video footage spans 43.13 h, recorded using a Sony FDR HD camera (Sony Corporation, Tokyo, Japan) with a resolution of 1280 × 720, a frame rate of 30 frames per second, and a sampling rate of 48 kHz.

In order to compare the differences in facial actions in different disease courses, two kinds of scenes—emotional elicitation and subjective elicitation—were collected, including 27 emotional corpora (9 sentences each of happy, sad, and angry emotion) and 9 neutral corpora. The former was induced by watching specific clips, while the latter was induced by self-selected situations based on subjects’ own experiences. During the assessment process, based on the Articulation Disorder Rating Scale and the patient’s specific presentation, clinical speech–language pathology specialists in the Rehabilitation Department classified patients into three severity levels: mild, moderate, and severe.

## 3. Multi-Level Labeling Method Based on Pre-Trained Model and Facial Motion Amplitude

**Figure 2:**
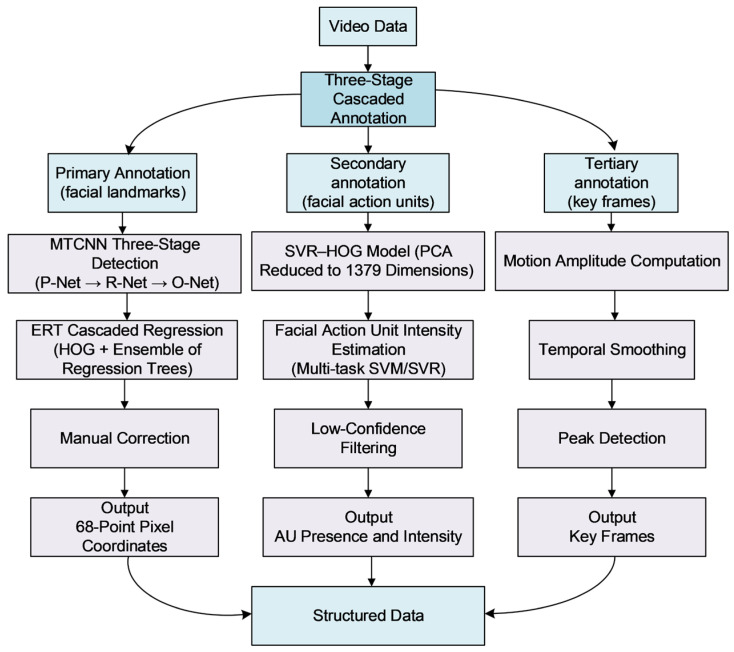
Illustration of the multi-level labeling framework based on pre-trained model and facial motion amplitude.

Automatic keyframe annotation heavily relies on labeled data and suffers from insufficient generalization capabilities. Existing keyframe annotation techniques fail to cover facial landmarks, facial action units, and keyframes. This study proposes a three-tier cascaded semi-automatic annotation framework tailored for video modalities. By integrating hierarchical collaborative automated algorithms with auxiliary verification, it efficiently completes annotations for facial landmarks, facial action units, and keyframes. The three-tier cascaded annotation framework is illustrated in Figure 2.

Level-1 annotation employs the Multi-Task Cascaded Convolutional Networks—Ensemble of Regression Trees (MTCNN-ERT) joint model for 68-point facial landmark detection. By integrating coarse localization via convolutional networks with fine-tuning through regression tree ensembles, it achieves optimal accuracy while maintaining real-time performance. Level-2 annotation utilizes the Support Vector Regression—Histograms of Oriented Gradients (SVR-HOG) pre-trained model for AU intensity estimation. It addresses data imbalance through Principal Component Analysis (PCA) dimensionality reduction, and multi-task learning, while automatically filtering low-confidence samples. Tertiary annotation introduces a keyframe extraction algorithm based on facial motion amplitude. By modeling and smoothing spatio-temporal movements across different regions, it precisely captures dynamic peaks in facial expressions.

### 3.1. Data Preprocessing

The proposed multi-level annotation system utilizes pre-trained models, such as MTCNN and SVR-HOG, to facilitate feature extraction. Nevertheless, given that these models are developed using standard facial datasets, they are susceptible to domain shift biases when deployed on dysarthric populations. This discrepancy arises largely from environmental variability and distinct pathological facial patterns that differ markedly from the healthy ‘norm.’ Consequently, to guarantee the reliability of facial feature analysis, we established a rigorous preprocessing pipeline.

#### 3.1.1. Data Slices

**Figure 3:**
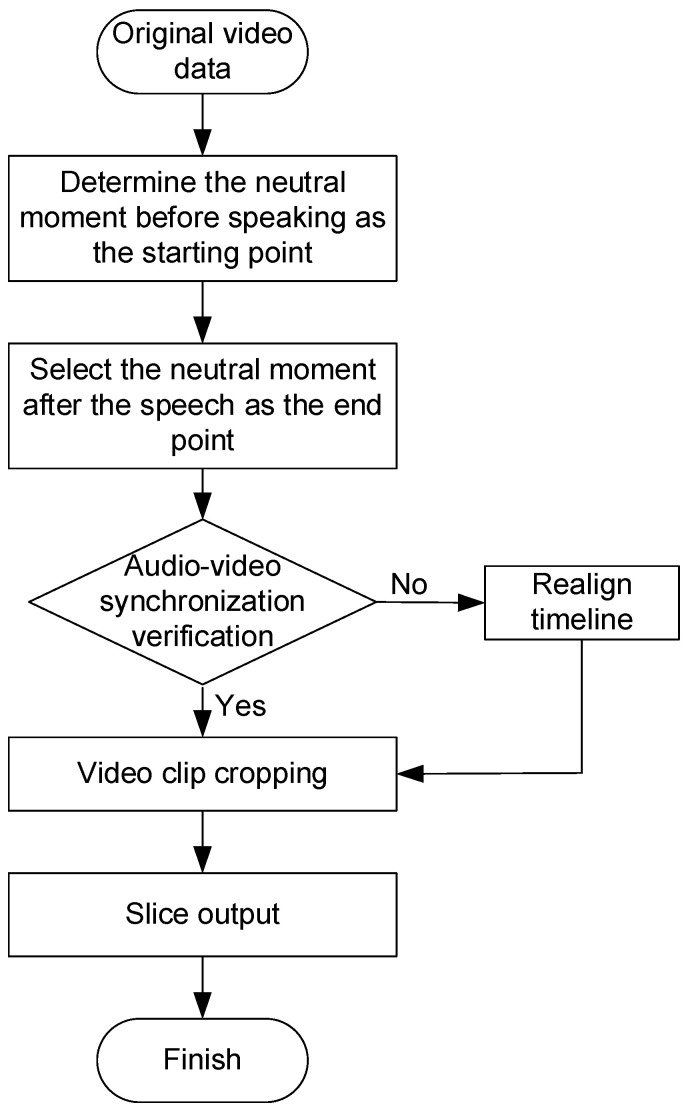
Video slice flow.

When processing video data, slicing is a critical step in data preprocessing. In order to ensure the accuracy and consistency of subsequent analysis, the slicing process needs to be carried out according to the corpus content and emotion change characteristics in the video. The video slicing process for this study is shown in Figure 3.

The starting point of the segment is selected at the neutral moment before the subject speaks, when the emotional state is stable and there is no obvious emotional fluctuation to avoid external emotional interference; the ending point is located at the moment when the subject speaks and the expression gradually returns to natural neutrality, so as to capture the whole process of emotional expression completely. During the slicing process, synchronization of speech and facial expressions is crucial, and it is necessary to ensure that the selected segments are strictly aligned in time to truly reflect the emotional state of the subject. In order to give consideration to the integrity of speech and expression fluctuations and the efficiency of subsequent processing, the segment duration is uniformly controlled between 2 and 6 s.

#### 3.1.2. Data Cleansing

**Figure 4:**
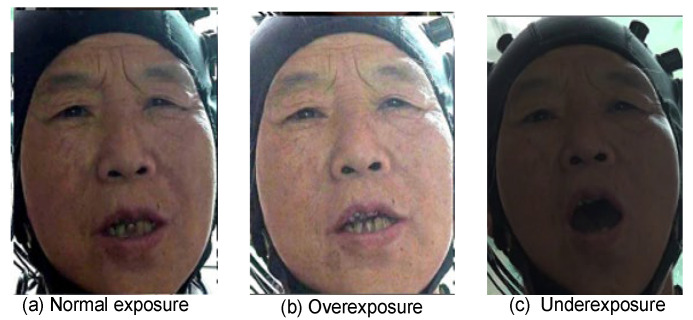
Video exposure inspection. All patients gave permission for the use of their images.

After video slicing is complete, data cleaning is required. Strict inspection was carried out to address the exposure problem and to remove the overexposed and underexposed video. As shown in Figure 4, Figure 4a is a well-lit video with good facial clarity, and Figure 4b and Figure 4c show overexposed and underexposed video, respectively. Over-exposure is due to brightness being too high in the video, resulting in white facial features, and under-exposure is due to insufficient light, resulting in blurred facial data.

To ensure each video has uniform exposure, this experiment adopts algorithmic detection followed by a second verification. First, we compute the luminance histogram of the video frames. For a grayscale frame $I\in R^{W\times H}$, its luminance histogram *H*(*b*) is defined as the number of pixels at each brightness level *b* ∈ [0, 255]. Here, *W × H* is the image resolution, and the total number of pixels is *N*_total_ = *W* × *H*.

(1) $$H(b)=\sum_{x=1}^{W}\;\sum_{y=1}^{H}\;\delta \left(I\left(x,y\right),b\right),\delta (a,b)=\{\begin{array}{cc} 1 & if\;a=b\\ 0 & otherwise \end{array}$$

**Figure 5:**
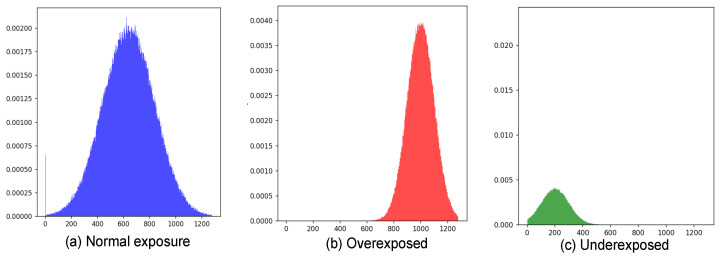
Calculated luminance histogram.

As shown in Figure 5, the luminance histogram of Figure 5a shows a uniform distribution, with luminance values covering areas from lower (dark) to higher (light), indicating that the image is balanced in terms of light and exposure. The right side of Figure 5b shows a large number of luminance values concentrated in higher areas, indicating that bright areas in the image are too bright. The left side of Figure 5c shows that the luminance values are concentrated in a low area, indicating that the dark area in the image is too dark.

Based on the statistical characteristics of the histogram, this study defines quantitative indices for overexposure and underexposure. Let the high-luminance threshold be $T_{\mathrm{high}}\;=\;250$; if Equation (2) is satisfied, the image is judged to be overexposed. Similarly, by setting a low-luminance threshold, if Equation (3) is satisfied, the image is judged to be underexposed. The detection results are then subjected to a second review to correct possible misjudgments.

(2) $$P_{high}=\frac{1}{N_{total}}\sum_{b=T_{high}}^{255}H\left(b\right)>5\%$$

(3) $$P_{low}=\frac{1}{N_{total}}\sum_{b=0}^{T_{low}}H\left(b\right)>10\%$$

According to the screening, there are 27 problem videos in total, 12 of which have overexposure problems and 15 of which have underexposure problems. In addition, in order to ensure accurate extraction and analysis of the subject’s facial features, it is necessary to check the facial occlusion in the video. In this study, Adobe Premiere Pro 2023 (version 23.6), a professional Media Processing Service tool, was used to examine 13 videos with obvious occlusion problems. The video with occlusion less than 10 frames was selected and retained, and video with occlusion exceeding 10 frames was eliminated.

### 3.2. Multi-Level Labeling Framework Based on Pre-Trained Model and Facial Motion Amplitude

The Multi-Task Cascaded Convolutional Network (MTCNN) [39] is used as the face detection model in this study. This model realizes efficient face detection, bounding box regression, and keypoint localization tasks through three cascaded convolutional neural networks: P-Net, R-Net, and O-Net. Firstly, the whole image is scanned quickly by P-Net to generate candidate face regions; then, R-Net further discriminates and regresses candidate frames, finely screens out false detections, and corrects boundary frames; finally, O-Net determines the final face boundary frames in the filtered regions and regresses five facial key points, and outputs the final face frames and key point coordinates.

We employed the cross-platform Dlib toolkit for 68-point keypoint annotation, with the core algorithm based on a cascaded regression framework combining directional gradient histogram feature extraction and Ensemble of Regression Trees (ERT). In ERT, each layer’s regression tree consists of *K* regression trees. The offset results from multiple trees are aggregated to form the final prediction. Each layer contains multiple regression trees, with each tree processing pixel difference features through binary split nodes.

The total offset S^(t)^ output by the Tth-level cascaded tree is

(4) $$\Delta S^{\left(t\right)}=\frac{1}{K}{\sum_{k=1}^{K}T}_{k}^{\left(t\right)}(x)$$

Among these, T_K_(t) denotes the kth regression tree at layer t, and represents the offset of the predicted facial keypoints at layer t. The final shape update formula is

(5) $$S^{\left(t+1\right)}=S^{\left(t\right)}+\Delta S^{\left(t\right)}$$

Starting with “initializing the average shape,” the system predicts offsets through layer-wise regression trees, continuously adjusting the positions of facial landmarks, and ultimately outputs a highly accurate prediction result of 68 key points.

### 3.3. Facial Action Unit Annotation Based on the SVR-HOG Pre-Trained Model

Facial action units (AUs) are detected and analyzed based on the Facial Action Coding System (FACS) [40], which decomposes facial expressions into 44 independent or coordinated muscle movement units. Table 1 lists the meanings of some AUs. Each AU represents an anatomically separable muscle movement with observable skin deformation characteristics.

**Figure 6:**
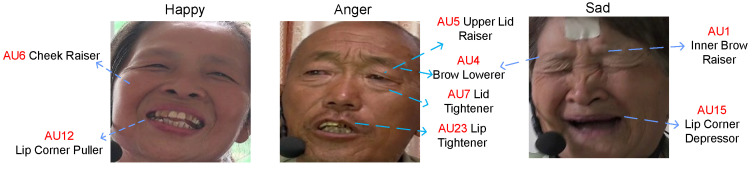
Associated facial action units under different emotions. All patients gave permission for the use of their images.

According to the correspondence between emotion and AU, we use and design algorithm to detect facial action units under their corresponding emotions. Figure 6 shows facial action units under happy, angry, and sad emotions.

**Figure 7:**
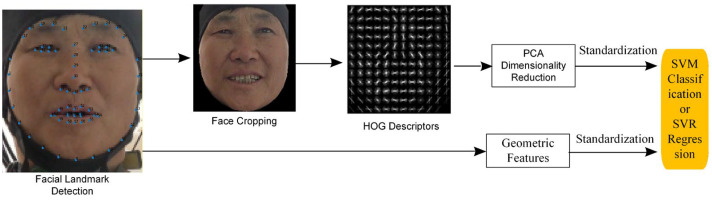
SVR-HOG labeling flow chart. The patient gave permission for the use of their images. All patients gave permission for the use of their images.

In this study, SVR-HOG pre-training model [41] is used to label the facial action units of the subjects. This model integrates the action unit detection and intensity estimation methods of Support Vector Regression (SVR) and Histogram of Oriented Gradient (HOG) features. A specific flow chart is shown in Figure 7. HOG features, also known as appearance features, are used for detailed texture changes, mainly describing visual modes such as facial texture, edges, and shadows. Geometric features are constructed based on the position of facial landmarks (such as corners of eyes and corners of mouth) to assist in detecting structural changes caused by AU movements, and these two are combined to predict AU occurrence and intensity.

First, facial landmarks are detected in the input image of the patient’s face, and geometric and HOG features are extracted from the standardized facial region. Next, Principal Component Analysis (PCA) is applied to the HOG features to reduce their dimensionality, lowering it from 4464 to 1379. This is followed by normalization processing. Meanwhile, geometric features are extracted based on keypoint coordinates to capture local deformations of facial action units, and then standardized. Finally, each set of features is input into Support Vector Regression (SVR) and Support Vector Machine (SVM) models for AU presence detection and intensity estimation, respectively.

AU detection establishes the presence of a specific AU (e.g., AU04, eyebrow elevation; AU12, corner of mouth elevation) in the current frame. This method uses an SVM classifier to output an activation status (0 or 1). AU intensity estimation uses an SVR regressor to predict the magnitude of AU movement and denote intensity levels on a scale from 0 to 5.

### 3.4. Key Frame Annotation Based on Facial Movement Amplitude

Video data consists of a large number of frames, which makes frame-by-frame analysis costly and inefficient. Keyframe annotation technology reduces data redundancy by selecting representative frames. Facial action amplitude measures the displacement of feature points relative to a neutral state, reflecting the magnitude and range of facial movements. This paper therefore proposes a keyframe annotation method based on facial action amplitude.

In order to establish a neutral reference baseline for facial states, this paper extracts five frames at equal intervals from videos depicting neutral emotions to form a reference frame set. As the research encompasses multiple emotional tasks, frame sequences during different expression transitions must be analyzed to extract key frames. To detect frame-level saliency, this study measures the displacement of key points in the regions of the left and right eyes, the nose, and the mouth. Table 2 shows the measured motion amplitude of each region based on the reference frame, which is used to quantify the local dynamic changes in the face.

The motion amplitude of each frame image is calculated relative to the first frame, based on that in the reference frame. First, the geometric displacements of key points in the left eye, right eye, nose, and mouth regions are computed and normalized. The relative rate of change is then defined separately for each region:

(6) $${∆}_{t}^{X}=\frac{\left|L_{t}^{X}-L_{0}^{X}\right|}{L_{0}^{X}}$$

Here, *t* denotes the index of the current frame; $L_{t}^{X}$ is the length of the feature vector for region x in frame t; $L_{0}^{X}$ is the length of the feature vector for the corresponding region in the reference frame, where X ∈ ${∆}_{t}^{X}$ denotes the normalized motion magnitude of region x in frame t relative to the reference frame.

The average relative change intensity across the four regions is then taken as the indicator of overall motion amplitude for the current frame. This serves as a measure of facial action intensity in that frame. The overall motion amplitude for frame t is as follows:

(7) $$Amplitude(t)=\frac{1}{4}({∆}_{t}^{nose}+{∆}_{t}^{mouth}+{∆}_{t}^{left\;eye}+{∆}_{t}^{right\;eye})$$

**Figure 8:**
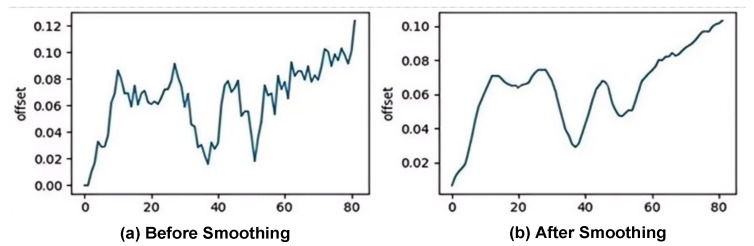
Smoothing of the video.

To reduce the noise influence existing in feature detection, this paper smooths the extracted facial motion amplitude sequence and further eliminates the frames with insufficient smoothing effect in the initial and last 30%. Figure 8 shows the changes in the offset curve before and after smoothing processing, where the horizontal axis represents the frame sequence number (frame index) and the vertical axis represents the offset. (a) represents the original motion amplitude sequence, which is subject to significant fluctuations due to the detection noise. (b) As a result of the smoothing process, the high-frequency noise is effectively suppressed while the main motion trend is retained. After smoothing, the curve fluctuations weaken and the overall trend becomes more stable.

### 3.5. Face Alignment Processing and Verification Based on Grayscale Normalization and Geometric Constraints

**Figure 9:**
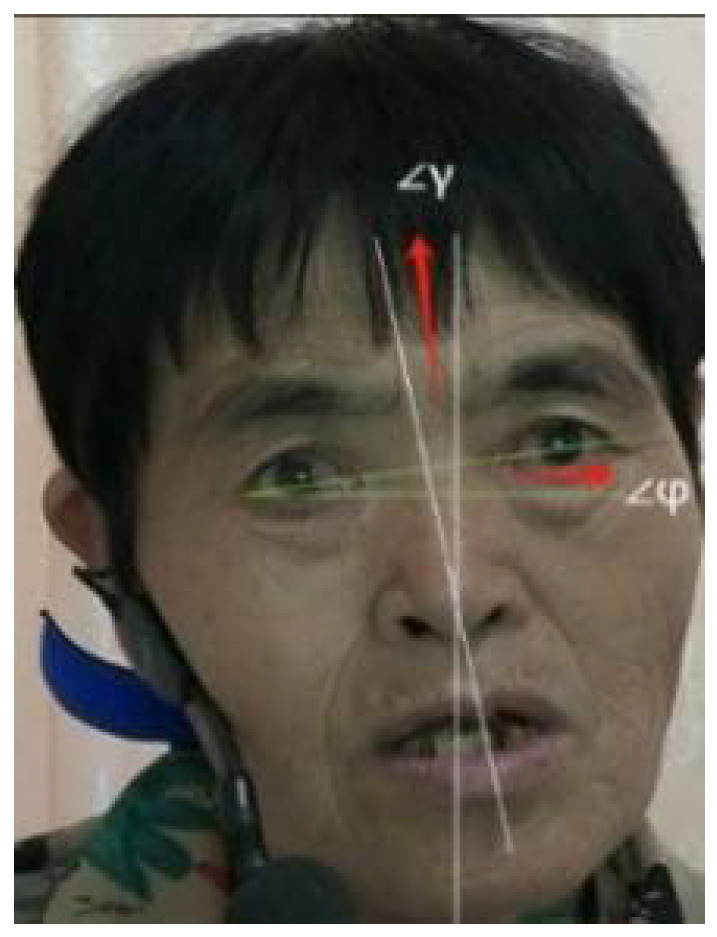
Face alignment.

In order to improve the accuracy and robustness of subsequent facial feature extraction and classification tasks, this paper first performs grayscale normalization processing on the extracted video frame images to reduce the interference caused by the illumination differences between different frames. Subsequently, the detected face areas are uniformly scaled to a fixed size of 200 to ensure the consistency of the data scale. On this basis, in order to further eliminate the influence of facial posture differences, alignment operations are performed on the standardized face images to center all faces and ensure that both eyes are on the horizontal line of the image. As shown in Figure 9, the angle between the line connecting the center of the eyes and the horizontal line is defined as *φ*, and the angle between the bridge of the nose line and the vertical direction of the image is defined as γ. These are used to describe the horizontal and vertical offset angles of the face, thereby evaluating the alignment effect.

In addition, the horizontal and vertical angle changes after face alignment were calculated from 20% of the data that was randomly selected to verify the accuracy of alignment, as shown in Table 3. The comparison results show that the mean and variance of the intersection angle after alignment are significantly reduced in most subjects, indicating that the alignment of the eyes and the bridge of the nose has achieved good standardization, and the facial posture tends to be consistent. However, in some individual subjects (such as m16 and m08), the angle values increased after alignment, due to minor deviations in keypoint positioning, significant differences in individual facial structures, or fluctuations in angles between frames. Overall, the alignment process effectively enhances the geometric consistency of facial images, providing a stable foundation for subsequent feature extraction and analysis.

### 3.6. Validity Analysis of Key Frame Labeling Methods

This study conducted experiments on the Spontaneous Actions and Micro-Movements Dataset (SAMM) [42] and Chinese Academy of Sciences Micro-Expression II (CASME II) [43] datasets to evaluate the effectiveness of keyframe detection based on facial motion amplitude. These datasets feature keyframe annotations. The average error margin of keyframe detection based on facial motion amplitude was then compared with that of several keyframe estimation methods developed in recent years.

SAMM consists of 32 subjects from mixed-race backgrounds. Emotion-evoked samples of 159 authentic microexpressions were collected, each lasting between 0.5 and 1.5 s. The dataset accurately labeled the beginning and end frames of each microexpression. CASME II includes 26 Chinese subjects, and 247 natural microexpression samples were collected through emotional video induction. These samples last between 0.5 and 2 s, and the vertex frames were marked as key frames targeting Asian people.

This experiment uses mean absolute error (MAE) as the evaluation metric. MAE is defined as the average frame error between the predicted and true keyframes:

(8) $$MAE=\frac{1}{N}\sum_{i=1}^{n}|e_{i}|$$

Here, $e_{i}$ is the error between the measured key frame and the ground-truth key frame for the i-th sample, and N is the number of samples in the test dataset.

As shown in Table 4, our method achieves an MAE of 11.6 and 12.5 on the datasets CASME II (66.9 frames) and SAMM (74.3 frames), respectively. In contrast, the THE-POSSD dataset features significantly longer sequences with slower motion dynamics, averaging 122.4 frames. Despite this nearly two-fold increase in duration, the MAE on THE-POSSD remains stable at 12.8 frames, representing a relative temporal error of 10%. These findings suggest that the proposed method mitigates error accumulation in longer sequences, supporting its potential applicability to the slower, heterogeneous facial motions characteristic of dysarthria.

After preprocessing the THE-POSSD video data, 2155 video clips were ultimately retained. These clips fall into four emotional categories: happy, sad, angry, and neutral. The dataset includes data from 60 individuals, including mild, moderate, and severe patients, as well as healthy controls. For each subject, 68 facial landmarks, facial action units, and five key frames per video were annotated through the experiments described in this section, as shown in Table 5.

## 4. Analysis of Facial Features in Patients with Dysarthria

### 4.1. Statistical Analysis of Facial Action Unit

In order to study the facial movement characteristics of dysarthria patients under different emotional tasks, this study relied on the pre-trained model SVR-HOG proposed in Section 3 to extract the existence and intensity scores of facial action units (AUs), and quantified the characteristics of the video sequences of each subject. The above score data constitute the facial action unit features (FAUF) used in this study. By calculating statistical features of FAUF and comparing among groups, the differences in facial motor patterns between dysarthria patients and healthy individuals were revealed.

**Figure 10:**
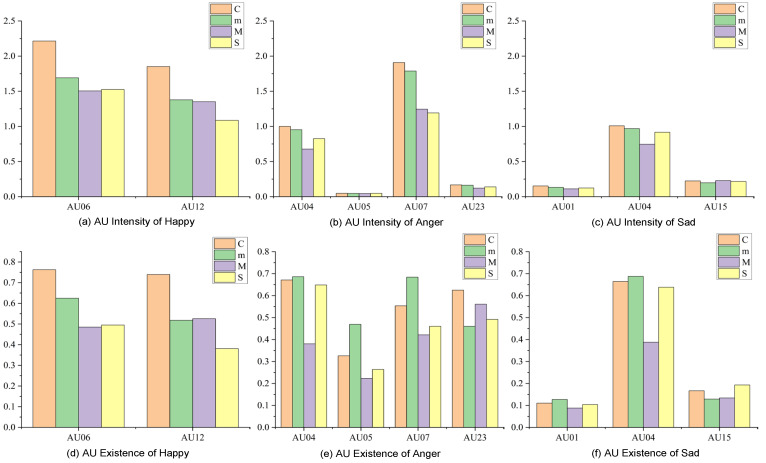
Comparison of AU intensity and existence mean of three emotions under different disease courses (in the figure, C denotes the normal control group, while m, M, and S represent patients with mild, moderate, and severe dysarthria, respectively).

First, the mean intensity and existence scores of each subject’s videos are calculated and analyzed. Figure 10 shows the comparison results of the mean characteristics between patients at different stages and control groups under three emotional tasks of happy, angry, and sad. It should be noted that since the existence degree of facial action units is binarized (0 indicates absence, 1 indicates presence), the result obtained by calculating the mean reflects the occurrence probability of the corresponding AU unit in the population.

On the whole, the AU of sad emotion (Figure 10c) is less activated in terms of intensity than the AU of happy and angry emotion (Figure 10a,b), with intensity values less than 1. In particular, the intensity values of AU01 (inner brow lift) and AU15 (mouth corner depression) (Figure 10f) are about 0.2, and their existence degrees are also low, about 0.2 or less. Under sad mood, the motion amplitudes of inner eyebrow lifting and mouth pressing are small, which makes the labeling algorithm based on motion amplitude unable to capture the displacement. Meanwhile, the intensity and presence of these two AUs (Figure 10c,f) are basically consistent between normal people and patients with different diseases. AU04 (eyebrow depression) is differentiated, with mild and moderate patients showing a decrease in the intensity of eyebrow depression compared to normal subjects, while severe patients also showed a decrease but a higher intensity than moderate patients (Figure 10c). The same phenomenon occurs in anger (Figure 10b). These findings suggest that severe stroke patients are more likely to activate eyebrow depression when angry or sad, are more sensitive to and experience persistent negative emotions, and more depressed than moderate stroke patients. As can be seen in the presence of anger and sadness (Figure 10e,f), the frequency of AU04 occurrence is relatively high, especially for anger, for which the probability value of eyebrow depression is about 0.65, which exceeds the probability of moderate patients (about 0.4) by about 25%. This shows that for mild and moderate patients, their emotions are relatively stable, and although they can reasonably control their emotions, this also leads to corresponding muscle weakness.

Severe patients, however, remain highly sensitive to negative emotions due to reduced emotional control, and can frequently mobilize muscle activity in relevant areas, especially around the eyes, such as AU04 and AU07 (eyelid tightening) (Figure 10b,e). AU07 is still the main action unit representing anger emotion, and although its activation intensity decreased with the increase in disease severity, the overall existence degree remained high. A similar pattern occurs in happy moods (Figure 10a,d), where the intensity and presence of AU06 (cheek lift) and AU12 (mouth lift) both decrease with severity, suggesting a limited ability to express emotion and a corresponding weakening of muscle activity.

The above analysis suggests that patients with severe stroke exhibit heightened sensitivity to negative emotions, as indicated by increased activity in the muscles around the eyes. This finding is consistent with clinical observations of post-stroke patients. Furthermore, the sensitivity of periorbital and perioral motor units to disease severity assessment makes them suitable for grading this condition.

To further explore the aforementioned differences, this study analyzed the intensities of specific action units (AUs) under three emotions in different disease severity levels compared to a healthy control group, including AUs 4, 6, 7, and 12.

**Figure 11:**
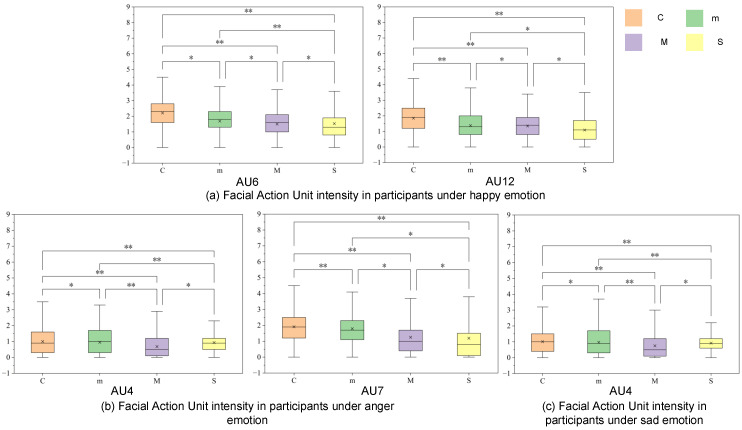
Boxplot of AU unit intensity under three moods for different disease courses (in the figure, C denotes the normal control group, while m, M, and S represent patients with mild, moderate, and severe dysarthria, respectively; “*” indicates *p* < 0.05, and “**” indicates *p* < 0.01; Bonferroni correction was applied).

Figure 11 presents box plots marked with statistical significance indicators. As shown in Figure 11a, AUs 6 (zygomaticus major muscle elevation) and 12 (orbicularis oris muscle) exhibited a gradual decreasing pattern (C > m > M > S). Paired statistical tests indicated significant differences between the healthy and diseased groups, as well as between adjacent severity stages (e.g., between the mild and moderate groups for AU12, *p* < 0.05). This suggests that dysfunction of the main zygomaticus muscle is associated with disease progression, supporting the use of AUs 6/12 to distinguish between mild and moderate dysarthria.

In the context of anger, the intensity distribution of AU7 (Figure 11b) also exhibits a similar downward trend. Mild patients maintain an intensity level comparable to the control group, while there is a significant decrease in the moderate and severe stages. Regarding AU4 (lower jaw depressor) during anger and sadness (Figure 11b,c), its distribution is non-monotonic. Patients in the mild and moderate stages exhibit lower intensity than the control group, while the intensity of severe patients is relatively higher compared to the moderate group. In AU4, the interquartile range (IQR) of severe patients is narrower than that of the control group and mild group. This degree of dispersion indicates homogeneity within the group. The significant difference (* *p* < 0.05) between the moderate and severe groups suggests that sensitivity to negative emotions distinguishes severe injuries from moderate cases. It is worth noting that this distinction is particularly evident in the context of sadness (Figure 11c), where the difference between the moderate and severe groups reaches a higher significance level.

In summary, the statistics and distribution analysis of FAUF quantify the differences in facial motor control across different levels of aphasia severity. The research results indicate a dissociation phenomenon in the muscle patterns around the mouth and eyes. The intensity of muscles around the mouth decreases in a stepwise manner, paralleling the progression of the disease, suggesting gradual muscle weakening. Conversely, in patients with severe conditions, the muscles around the eyes exhibit higher intensity levels and lower variability under negative emotions, which is different from patients with milder conditions, indicating a defect in inhibitory control. These results effectively reflect physiological dysfunction and emotional regulation deficits, providing objective measurement standards for clinical assessment and grading of dysarthria.

### 4.2. Construction of Multi-Region Facial Keypoint Graph Structure Based on Delaunay Triangulation

Traditional convolutional networks usually regard facial key points as having a regular grid structure, which makes it difficult to effectively characterize the non-Euclidean topological relations and local asymmetric motion features of dysarthria patients’ faces. To overcome this limitation, Delaunay-based Facial Landmark Graph Features (DFLGF) are proposed in this paper. By constructing anatomical associations between key points in multi-region graph structure modeling, the feature expression ability of abnormal regions is enhanced, and more discriminative representations are provided for subsequent disease classification tasks.

Delaunay triangulation connects a set of points to form a non-overlapping triangular network. By maximizing the minimum angle to avoid elongated triangles, it accurately captures the spatial adjacency relationships between key points, making it a popular choice for structural modeling and topological analysis. The principle behind it is as follows:

**Step 1:** Empty-circumcircle property. For a set of planar points $P=\{p_{1},p_{2},\ldots ,p_{n}\}$, the set of triangles $T=\{t_{1},t_{2},\ldots ,t_{m}\}$ produced by the Delaunay triangulation must satisfy

(9) $$\forall t_{i}\in T,\;C\left(t_{i}\right)P_{j}\in P\left(j\neq i\right)$$

**Step 2:** Delaunay triangulation maximizes the smallest angle of the resulting triangle among all possible triangulations.

**Step 3:** For any quadrilateral composed of two adjacent triangles, if these two triangles do not satisfy the property of an empty circle, the triangulation can be optimized by an edge flip operation.

In order to investigate the contribution of different facial regions to disease classification, a multi-region graph structure was constructed. Decoupling the dynamic characteristics of sensitive facial movement areas (such as the lips, nasolabial folds, eyebrows, and eyelids) allows the contribution weights of different regions to disease classification to be quantified. The specific implementation is as follows:

**Step 1:** Four types of feature maps are constructed based on the coordinates of 68 key points. Eyebrow keypoints are numbered 17–28, eyes 36–47, mouth 48–68, and full face 17–68, where full face represents keypoints in the eyebrow, eyes, nose, and mouth regions.

**Figure 12:**
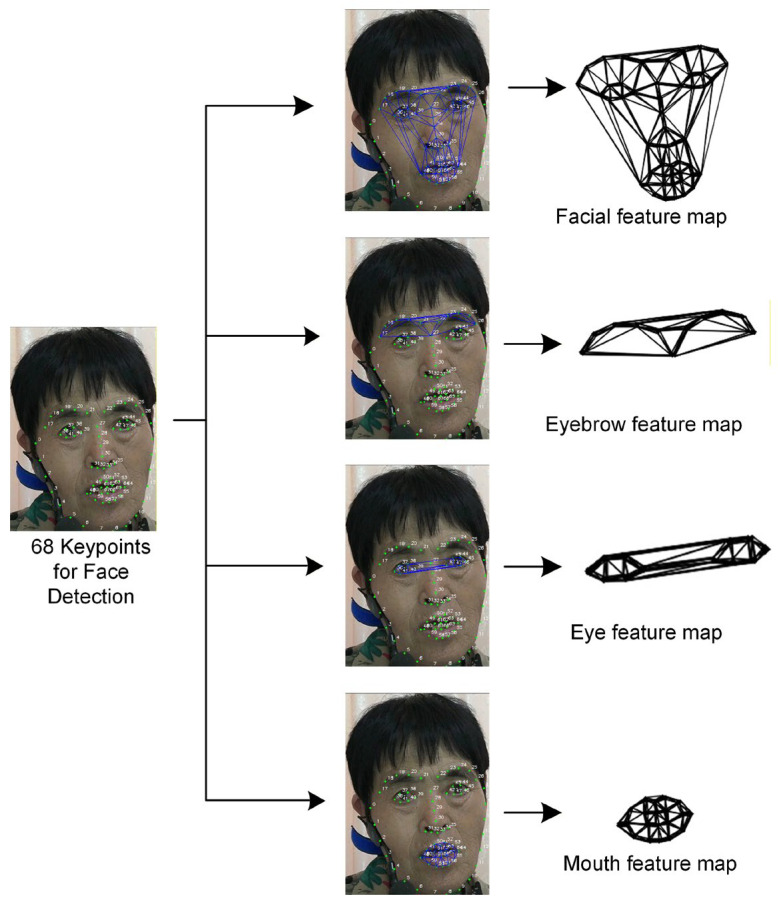
Constructing feature maps using Delaunay triangulation.

**Step 2:** A face map structure is constructed based on Delaunay triangulation. Specifically, the system locates 68 key points in the face image, divides the face into four sub-regions (whole face, eyebrows, eyes and mouth) according to functional regions, and constructs the local map structure by Delaunay triangulation for each key point. This construction process is illustrated in Figure 12.

These graph structures not only preserve topological relations, but also describe the motion patterns of the eyebrows, eyes, and mouth. The map representations are then converted into GNN usable inputs as the basis for structured visual features for facial anomaly recognition, emotion analysis, and dysarthria assessment. This method integrates global and local dynamics and strengthens cross-regional collaborative modeling.

### 4.3. Validity Analysis of Key Points in Different Facial Regions

To explore the contribution of facial keypoints extracted in Section 4 to dysarthria severity grading, DNNs were used to analyze the effectiveness of the eyebrows, eyes, mouths, and whole faces of dysarthria patients. As shown in Table 6, the experimental results show that each face region exhibits good classification performance.

With an accuracy of 85.1% and an F1 score of 85.3%, the eye region outperformed the eyebrow and mouth regions. This can be attributed to the higher motor specificity exhibited by the periorbital muscle group in articulation disorders. The performance of the full-face feature significantly surpassed that of any single region, indicating that facial abnormalities in articulation disorders exhibit global characteristics. Furthermore, there are complementary relationships among features across different regions, and integrating multi-regional information improves classification performance.

This study used a graph structure based on Delaunay triangulation to divide facial landmarks into different regions. A quantitative analysis was then performed on each region to determine its discriminative capability. The results demonstrate that this approach effectively identifies the critical importance of the eye and perioral muscle regions when grading the severity of articulation disorders. This validates the feasibility and efficacy of using facial landmark features in clinical assessments.

### 4.4. Validity Analysis of FAUF and DFLGF

In order to verify the validity of facial action unit features (FAUF) and Delaunay-based Facial Landmark Graph Features (DFLGF) for classification of dysarthria patients, this section uses Support Vector Machine (SVM) and deep neural networks (DNNs) as basic networks to classify dysarthria patients. Table 7 shows the performance of facial action unit features and facial keypoint features under SVM and DNN classification models.

Overall, both feature sets achieved a classification accuracy greater than 80%. In terms of feature dimensions, DFLGF demonstrated superior recognition performance to FAUF, suggesting that it more directly reflects the unique facial movement abnormalities and asymmetry characteristic of patients with dysarthria. Furthermore, when both features were combined, SVM achieved an accuracy of 84.0%, while DNN reached 88.8%. This suggests that FAUF and DFLGF complement each other well, and using them together can provide a more comprehensive characterization of the facial abnormalities associated with dysarthria. The method comparisons revealed that DNN consistently outperformed SVM across all feature combinations, particularly when using DFLGF and the merged features. This demonstrates the superior capability of deep neural networks in processing and integrating complex, high-dimensional, nonlinear features. Consequently, deep learning-based multimodal feature fusion methods can significantly improve the accuracy of dysarthria classification.

## 5. Classification of Dysarthria Based on Facial Visual Features Compensating Acoustic Features

### 5.1. The Proposed VACP-Net Framework

In order to compensate for the deficiency of the single acoustic mode in the identification of dysarthria, the visual mode is introduced to enhance the overall representation ability of pathological features. Specifically, a multimodal feature set is constructed by fusing facial action unit features (FAUF), facial keypoint map features (DFLGF), and acoustic features to verify the compensation effect of visual features on acoustic modalities and their effectiveness in disease classification.

**Figure 13:**
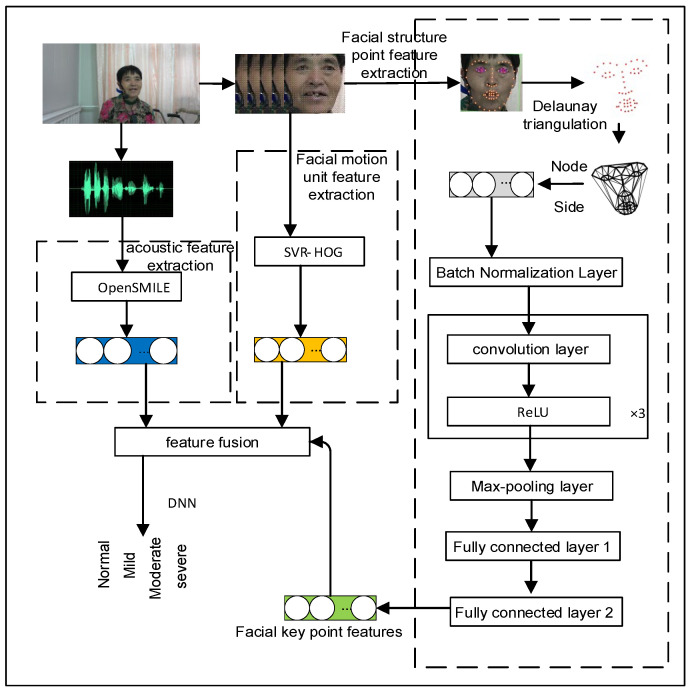
VACP-Net network architecture.

This study proposes a technical framework for multimodal feature set fusion of VACP-Net (Visual–Acoustic Compensatory Pathway Network), as shown in Figure 13. The framework model consists of two modules: feature module and classifier module. Among them, the feature module includes the facial action unit feature (FAUF), facial keypoint feature (DFLGF), and acoustic feature (eGeMAPS) sets [49].

The facial action unit features (FAUF) extract a 35-dimensional characterization vector from each frame of video, which includes 18 AU unit strength regression values and 17 AU unit existence logic values, corresponding to facial action unit strength and existence values, respectively.

Facial keypoint features (DFLGF) firstly construct a graph structure based on the aligned facial key points by using the Delaunay triangulation method to conform to the spatial connection relationship of facial anatomical structure, and takes the Euclidean distance between key points as the weight of edges in the graph. The feature map is then input into a graph convolutional network (GCN) to encode local and global spatial dependencies between key points and extract high-level features. Finally, after maximum pooling and fully connected layer processing, the features are mapped to 128-dimensional facial keypoint representation vectors.

Acoustic features are the core of speech processing. Extraction and analysis of acoustic signals from patients can identify dysarthria pathology, train high-precision classification models, assist clinical classification, and provide timely treatment recommendations. For speech data from dysarthria patients, this study uses the OpenSMILE toolbox to extract the eGeMAPS feature set, which is used to compare and analyze the feature performance of acoustic channels. This feature level contains 88 features, covering frequency, energy, spectrum, and other aspects, and can comprehensively describe the acoustic characteristics of speech signals. See Table 8 for details.

Then, the facial action unit features (FAUF), facial keypoint features (DFLGF), and acoustic features (eGeMAPS) are fused at the feature level to form a 251-dimensional joint feature vector, as shown in Table 9, and these feature vectors are input into DNN for disease classification.

To improve the efficiency of feature fusion and the performance of the hierarchy, this study uses a staged feature processing strategy. First, the features of facial action units and keypoints are optimized and extracted independently. Cross-modal feature fusion is then achieved at higher levels, fully leveraging the discriminative advantages of the different modalities.

### 5.2. Experimental Setup

All experiments in this study were conducted on a 64-bit Windows 11 platform (Microsoft Corporation, Redmond, WA, USA) equipped with an Intel Core i7 processor (Intel Corporation, Santa Clara, CA, USA), 16 GB of memory, and an NVIDIA GeForce RTX 3050 graphics card (NVIDIA Corporation, Santa Clara, CA, USA). The programming environment was Python 3.10.

In terms of model parameter configuration, the Support Vector Machine (SVM) used the Radial Basis Function (RBF) as the kernel function, with the penalty factor C set to 4 and the kernel function coefficient g (gamma) set to 0.25. The deep neural network (DNN) constructed a network architecture with 4 hidden layers, with the number of neuron nodes in each layer being 128, 64, 32, and 4 respectively. The learning rate in the optimization process was set to 0.01.

To evaluate the robustness of the model and ensure the reliability of the experimental results, the study adopted a repeated random partitioning validation strategy [50]. Specifically, the dataset was randomly divided into a training set and a test set in a 8:2 ratio, and stratified sampling was used to maintain the distribution of classes after partitioning, consistent with the original data. This process was repeated for 5 independent experiments by setting different random seeds, and the final performance indicators (accuracy and F1 score) reported in the text are the average values of these 5 experiments.

### 5.3. Analysis of Experiment Complexity and Efficiency

In order to evaluate the computational efficiency and reasoning performance of the system in a standard experimental environment, this section calculates the total time required for the entire inference process from the preprocessing of the original multimodal input (visual and auditory paths) to the final model.

Table 10 shows the core components of the VACP-Net model and the end-to-end inference time. Although the preprocessing and feature extraction steps account for the majority of the total inference time, the end-to-end processing time of the system still remains stable within 4.5 s, demonstrating excellent real-time processing potential. Additionally, the total number of parameters of the entire model is approximately 50,000, and the floating-point operation count (FLOPs) for a single inference is 0.1 G, indicating good lightweighting and computational efficiency.

### 5.4. Comparison Experiment and Analysis Between Channels

In the inter-channel contrast experiment, the disease classification of different methods in the acoustic channel, image channel, and acoustic and video fusion channel is analyzed in detail, and the results are shown in Table 11.

In the acoustic channel experiment, the random forest model achieved classification accuracies of 74.8% and an F1 score of 75.0%. By contrast, the deep neural network (DNN) model demonstrated superior performance, improving the accuracy and F1 score to 78.6% and 77.7%, respectively. These results suggest that DNNs are more effective at capturing nonlinear and temporal interactions in acoustic features, resulting in greater precision and robustness. These findings further validate the advantages of deep learning in feature extraction and classification for acoustic modalities.

In the image channel experiment, the graph structure features (DFLGF) constructed based on Delaunay triangulation and the graph convolutional network (GCN) show the superior ability of disease recognition, and the accuracy rate and F1 score of the model reach 88.9% and 88.8%, respectively, under this setting, which verifies the effectiveness of the optimized graph structure in capturing the spatial relationship of facial key points. The accuracy and F1 score of the model reach 90.3% and 90.1% respectively, which indicates that AU feature provides complementary discriminant information in facial motion pattern expression, which is helpful to enhance the classification effect of visual modality in dysarthria severity classification.

**Figure 14:**
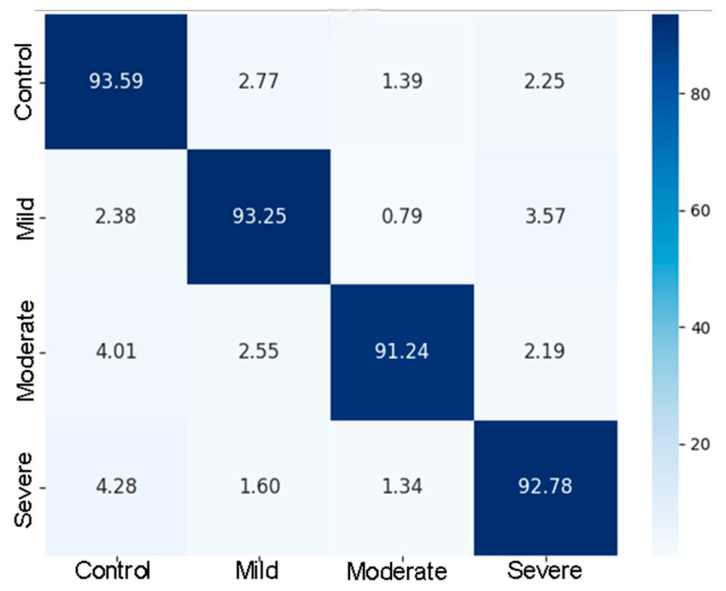
VACP-Net confusion matrix.

To further verify the above conclusions, the confusion matrix in Figure 14 shows that the overall accuracy of the model in the four-classification task is high, with more than 91% of the samples in all four categories. Normal subjects (93.59%) and mild patients (93.25%) were the most stable, and the misclassification rate was low, indicating that the model had a strong discrimination ability between healthy samples and early lesions. 4.01% and 4.28% of moderate and severe patients were misjudged as normal persons, respectively, reflecting that moderate and severe patients had unstable speech expression, some of them had normal pronunciation, and some had an obvious disorder. Combined with the analysis in Section 4, this instability was associated with decreased AU activation levels and consistency in facial expression tasks with disease.

### 5.5. Comparison Experiment with Other Methods

In order to ensure the fairness of the comparison, all the comparison models were re-implemented and trained based on the THE-POSSD dataset. We maintained the exact data division method and preprocessing procedure as those of VACP-Net. For BDCNN and MER-GCN, we made migration adjustments according to the parameter settings in their original papers to adapt to the input dimensions of this dataset. Table 12 shows the final evaluation results of each model on the same test set.

In the classification task of dysarthria, CNN and ResNet18 find it difficult to effectively characterize the complex nonlinear features in patients’ facial expressions and acoustic signals due to their limited feature modeling ability, resulting in limited room for classification performance improvement, with accuracy rates of 85.2% and 86.4%, respectively. BDCNN effectively enhances the dynamic expression ability by introducing optical flow information fusion convolution features, and its accuracy and F1 score are improved to 89.8% and 89.6%, respectively. MER-GCN combined a residual module with an AU level feature extraction mechanism, achieving 88.4% accuracy and an 87.3% F1 score. It should be pointed out that although BDCNN fuses optical flow and convolution features, both belong to visual modalities; MER-GCN does not construct a face map structure and only relies on AU-level features for modeling.

By contrast, the proposed VACP-Net uses Delaunay triangulation to create facial graph structures and graph convolutional networks to identify spatial relationships. By combining facial features (FAUF and DFLGF) with acoustic features (eGeMAPS), it makes the most of complementary multimodal information, achieving the best performance of all the models, with an accuracy rate of 92.0% and an F1 score of 91.6%.

In summary, the VACP-Net method offers clear benefits in terms of integrating multimodal features and modeling complex nonlinear relationships. It provides an efficient and robust solution for classifying articulatory disorders.

## 6. Discussion

Dysarthria affects not only speech function but is also inextricably linked to facial motor control. Our investigation into facial dynamic characteristics revealed that the presence and intensity of periocular and perioral motor units decrease significantly with disease severity. These findings highlight the potential of these regions as sensitive markers for disease classification, aligning consistently with clinical observations regarding the degradation of the orbicularis oris and orbicularis oculi muscles [53,54,55]. Beyond physiological markers, this study validates the critical role of multimodal fusion. While most existing studies focus predominantly on acoustic analysis, they often overlook facial dysfunction caused by hypomimia (lack of expression). By introducing visual features as a compensatory modality, we observed a significant improvement in classification accuracy. These results suggest that visual features not only compensate for the loss of nonverbal information in acoustic modes but also enhance the model’s sensitivity to pathological changes, underscoring the potential of multimodal fusion in clinical auxiliary diagnosis.

Capturing these pathological features requires robust labeling methods. Existing approaches mostly adopt single-structure frameworks, such as regular grid-based convolutional networks or temporal modeling networks. While suitable for healthy subjects, these methods struggle with the heterogeneity of facial structures and the weak muscle activation patterns typical of dysarthria patients, often resulting in insufficient pathological feature representation. To address this, we proposed a multi-level labeling method that integrates keypoint localization, action unit recognition, and keyframe extraction. By taking into account multi-task collaboration, this approach enhances the representation of abnormal motion in the spatio-temporal dimension, providing more robust feature support for subsequent classification and evaluation compared to traditional static or single-task methods.

Given the inherent differences in the evaluation paradigms of clinicians and models, clinical assessment—though the primary diagnostic basis rooted in professional experience—suffers from subjectivity, which leads to compromised consistency due to physician fatigue and inter-rater variability (consistency coefficients: 0.70–0.85 [56]). In contrast, the proposed VACP-Net (92.0% accuracy) features the objectivity inherent to automated tools and boasts internal consistency unaffected by fatigue or workload pressures. Its core value lies in acting as a complementary auxiliary tool: it provides objective, quantifiable references to alleviate clinicians’ diagnostic burden and mitigate judgment fluctuations, and we will further optimize and iterate the model in future work to better meet clinical auxiliary needs.

The experimental data in this study was acquired using a standard Sony FDR camcorder with a resolution of 1280 × 720 (30 fps) and a sampling rate of 48 kHz. In terms of computational efficiency, we employed pre-trained models and conducted rigorous evaluation. The results demonstrate that high accuracy is maintained with a lightweight architecture. Specifically, our quantitative analysis shows that the VACP-Net model requires 43.4 K parameters and 0.08 MFLOPs. Although the feature extraction pipeline takes ~4.5 s on a Windows 11 system equipped with an Intel i7 processor, 16 GB RAM, and an NVIDIA GeForce RTX 3050 GPU, the ultra-fast inference (~2 ms) ensures a total end-to-end latency of under 5 s per patient, confirming that this method has low requirements for computing resources.

Dataset size is a prevalent challenge in dysarthria research. However, compared to existing resources, the Whitaker database [21] solely encompasses acoustic modality data, with a total of 10 subjects, whereas the Torgo database [22] offers both acoustic and phonatory motor multimodal data (comprising 15 subjects in total, including 8 patients with dysarthria and 7 healthy controls), but its research primarily focuses on the correlation between physiological mechanisms and acoustic manifestations, and it does not include facial video data. The DEED [23], despite covering audio–video multimodal information, only includes four subjects, resulting in a limited sample size. In this context, our THE-POSSD dataset presents a comparatively substantial cohort of 60 participants (35 patients and 25 healthy controls), integrating acoustic, glottal, and facial video streams with emotional stimuli. To address the risk of overfitting common in this domain, we utilized pre-trained models for robust feature extraction and implemented Batch Normalization and Dropout for regularization. In addition, we also adopted a repeated random hold-out validation strategy to demonstrate the stability of the model.

Despite these measures, there are still some limitations. Firstly, although the sample size is relatively large for this field, it is still limited. Secondly, multimodal fusion methods need to be further improved in terms of robustness against environmental interference. Thirdly, regarding generalizability, the experimental dataset is derived from a single linguistic and cultural background. Although the core physiological manifestations of dysarthria (e.g., acoustic anomalies and facial muscle weakness) share structural commonalities across different populations, we recognize that linguistic differences may still exert a potential impact on model performance.

Building upon the facial motion feature annotation system established in this study, our future work aims to address these limitations while further bridging the gap in semantic-level analysis. Specifically, we plan to advance by constructing a more systematic and standardized multimodal dataset, with a particular focus on cross-language verification to enhance generalizability. Furthermore, we will attempt to combine Natural Language Processing techniques with Graph Neural Networks to explore the collaborative modeling of language, vision, and speech modalities. These efforts are designed to not only improve robustness against environmental and cultural variations but also to provide a reference for potential applications in clinical rehabilitation and personalized training scenarios.

## 7. Conclusions

This study proposes a robust multimodal imaging-driven framework for dysarthria severity classification, aiming to achieve objective and quantitative characterization of facial motor impairments. By integrating facial geometric representations derived from visual imaging with acoustic features, the proposed approach effectively compensates for the limitations of traditional unimodal assessment strategies. A multi-level annotation scheme is introduced to alleviate the reliance on large-scale expert-labeled data, while Delaunay triangulation combined with graph convolutional networks (GCNs) enables structured spatial–topological modeling of abnormal facial kinematics and muscle coordination. The experimental results using the THE-POSSD dataset demonstrate that the proposed VACP-Net achieves 92.0% accuracy, highlighting the significant contribution of visual pathological facial features to diagnostic performance. Moreover, the analysis of region-specific facial sensitivity under different emotional conditions provides imaging-based insights into the relationship between facial paralysis and impaired emotional expression. Overall, this work presents a promising imaging-oriented analytical framework for dysarthria assessment. Future studies will focus on validating the model on larger, independent cohorts to ensure clinical robustness, as well as enhancing cross-dataset generalization and extending the framework to real-time monitoring and broader neurological disorders.

## Figures and Tables

**Table 1:** Part AU meaning.

AU	Corresponding Facial Action	AU	Corresponding Facial Action
AU1	Inner Brow Raiser	AU9	Nose Wrinkler
AU2	Outer Brow Raiser	AU7	Lid Tightener
AU4	Brow Lowerer	AU12	Lip Corner Puller
AU5	Upper Lid Raiser	AU15	Lip Corner Depressor
AU6	Cheek Raiser	AU23	Lip Tightener

**Table 2:** Motion amplitude of four facial regions. Landmarks[x] in this table represent the coordinates of the xth key point, where x represents the corresponding keypoint index, and Dist(α, β) indicates the Euclidean distance between points α and β.

Facial Parameters	Formula
Nose center	landmarks[30]
Mouth center	(landmarks[48]+landmarks[54])/2
Left-eye center	(landmarks[36]+landmarks[39])/2
Right-eye center	(landmarks[42]+landmarks[45])/2
Nasal reference length	dist(nc,landmarks[27])+dist(nc,landmarks[31]) +dist(nc,landmarks[35])
Oral reference length	dist(mc,landmarks[48])+dist(mc,landmarks[54])
Left-eye reference length	dist(lec,landmarks[36])+dist(mc,landmarks[39])
Right-eye reference length	dist(rec,landmarks[42])+dist(rec,landmarks[45])

**Table 3:** Comparison of horizontal and vertical angles before and after face alignment.

Subject	*φ* Before (Mean, Var.)	*φ* After (Mean, Var.)	γ Before (Mean, Var.)	γ After (Mean, Var.)
C01	3.75 (28.58)	1.19 (0.88)	3.61 (26.79)	1.39 (6.07)
C02	5.84 (6.02)	1.41 (0.33)	4.46 (5.57)	2.22 (1.84)
C06	4.80 (10.25)	3.86 (5.47)	6.27 (10.92)	2.26 (4.17)
C12	9.55 (38.77)	5.30 (9.39)	1.06 (25.25)	4.97 (25.20)
C13	1.63 (3.11)	1.56 (2.30)	1.79 (4.68)	1.17 (5.27)
C16	4.33 (7.95)	3.01 (3.51)	6.39 (18.13)	7.48 (6.41)
m08	2.54 (8.34)	1.57 (3.88)	13.66 (7.81)	8.09 (14.58)
m16	3.13 (8.90)	3.94 (35.42)	8.41 (29.66)	6.50 (25.37)
M03	8.59 (2.10)	0.88 (1.10)	6.04 (2.17)	3.46 (2.42)
M04	2.79 (12.78)	0.89 (2.76)	1.05 (19.84)	2.14 (6.41)
S05	9.06 (57.40)	1.62 (6.37)	11.77 (66.46)	1.87 (13.88)
S13	1.89 (1.67)	1.21 (1.10)	1.26 (0.44)	2.52 (3.71)

**Table 4:** Comparison of key frame determination methods.

Method	Test Dataset	Avg. Sequence Length (Frames)	MAE (Frame)
DCNN [44]	CASME II	66.9	11.8
Bisection Search [45]	CASME II	66.9	13.6
Optical-Flow Features [46]	CASME II	66.9	11.8
HTNet [47]	SAMM	74.3	12.9
STA-GCN [48]	SAMM	74.3	12.7
Ours	CASME II	66.9	11.6
	SAMM	74.3	12.5
	THE-POSSD	122.4	12.8

**Table 5:** The THE-POSSD dataset after annotation.

Data Type	Subjects	Emotion States	Total	Annotations
Video data	Normal: 25	Happy	2155 clips	68 facial landmarks
	Mild: 13	Sad		
	Moderate: 11	Angry		AU (facial action unit) presence and intensity
	Severe: 11	Neutral		five key frames per clip at peak motion displacement

**Table 6:** Comparison of landmark-based features across facial regions.

Facial Region	Landmark Indices	Accuracy (%)	F1 (%)
Brows	17–28	84.5	84.2
Eyes	36–47	85.1	85.3
Mouth	48–68	84.2	84.3
Whole face	17–68	86.6	86.0

**Table 7:** Comparison of classification results based on FAUF and DFLGF.

Feature	Method	Accuracy (%)	F1 (%)
FAUF	SVM	81.6	82.0
	DNN	83.5	83.2
DFLGF	SVM	83.4	83.6
	DNN	86.6	86.0
FAUF + DFLGF	SVM	84.0	84.3
	DNN	88.8	89.1

**Table 8:** eGeMAPS feature set.

Category	Feature Types
Acoustic features	Fundamental frequency, energy, spectral features, formants, dynamic features
Statistical descriptors	Mean, standard deviation, maximum, minimum, range, slope, offset, squared error, skewness, kurtosis

**Table 9:** Feature sets and vector dimensionality.

Feature	Dimensionality
Facial action unit features (FAUF)	35
Facial landmark-based features (DFLGF)	88
Acoustic features (eGeMAPS)	128
Total	251

**Table 10:** The inference time of each component of VACP-Net.

Stage	Component		Latency (per Sample)
Preprocessing	Visual Pipeline	FAUF	~3.20 s
		DFLGF	~2.40 s
	Acoustic Pipeline	eGeMAPS	~0.20 s
Inference	VACP-Net Model	GCN (Visual Encoder)	~0.15 s
		DNN (Fusion Classifier)	~0.002 s
Total	End-to-End System		~3.60–4.50 s

**Table 11:** Comparison experiments between channels.

Channel	Method (Features + Classifier)	Accuracy	F1 (%)
Acoustic	eGeMAPS + Random Forest	74.8	75.0
	eGeMAPS + DNN	78.6	77.7
Video	DFLGF + GCN	88.9	88.8
	DFLGF + FAUF + GCN	90.3	90.1
Acoustic + Video	DFLGF + FAUF + eGeMAPS + Random Forest	81.2	81.4
	DFLGF + FAUF + eGeMAPS + DNN	92.0	91.6

**Table 12:** Comparison with other methods.

Model	Accuracy (%)	F1 (%)
CNN	85.2	85.3
Resnet18	86.4	85.7
BDCNN [51]	89.8	89.6
MER-GCN [52]	88.4	87.3
VACP-Net	92.0	91.6

## Data Availability

The data presented in this study are available on request from the corresponding author. The data are not publicly available due to privacy and ethical restrictions, as they contain sensitive multimodal information (facial videos and acoustic recordings) that could compromise the privacy of the participants.
